# Treatment of a mouse model of ankylosing spondylitis with exogenous sclerostin has no effect on disease progression

**DOI:** 10.1186/s12891-015-0823-8

**Published:** 2015-11-26

**Authors:** Katelin R. Haynes, Hsu-Wen Tseng, Michaela Kneissel, Tibor T. Glant, Matthew A. Brown, Gethin P. Thomas

**Affiliations:** The University of Queensland Diamantina Institute, Translational Research Institute, Princess Alexandra Hospital, Woolloongabba, Queensland Australia; Musculoskeletal Disease Area, Novartis Institutes for Biomedical Research, Basel, Switzerland; Section of Molecular Medicine, Department of Orthopedic Surgery, Rush University Medical Center, Chicago, Illinois USA

**Keywords:** Ankylosing spondylitis, Sclerostin, Wnt inhibitor, Mouse model, Bone formation

## Abstract

**Background:**

No treatment to date is available which specifically targets bone formation in ankylosing spondylitis (AS). Several recent studies have shown that sclerostin (SOST), a Wnt inhibitor specific to osteocytes and chondrocytes, is down-regulated in AS patients. This suggests Wnt signalling may be upregulated, and application of exogenous recombinant SOST (rSOST) may inhibit Wnt signalling and slow pathological bone formation.

**Methods:**

The proteoglycan-induced spondylitis (PGISp) mouse model in which we have previously demonstrated downregulated SOST expression, was used for this study. Mice were injected with 2.5ug rSOST/day for a period of 8 weeks following induction of disease. Axial skeleton disease development was assessed by histology and skeletal changes examined using DEXA.

**Results:**

rSOST treatment had no effect on peripheral or axial disease development, bone density or disease severity. Injected rSOST was stable over 8 h and residual levels were evident 24 h after injection, resulting in a cumulative increase in SOST serum levels over the treatment time course. Immunohistochemical examination of SOST levels within the joints in non-rSOST treated PGISp mice showed a significant decrease in the percentage of positive osteocytes in the unaffected joints compared to the affected joints, while no difference was seen in rSOST treated mice. This suggests that rSOST treatment increases the number of SOST-positive osteocytes in unaffected joints but not affected joints, despite having no impact on the number of joints affected by disease.

**Conclusions:**

Although not disease-modifying, rSOST treatment did appear to regulate SOST levels in the joints suggesting biological activity. Further dose response studies are required and SOST may require modifications to improve its bone targeting ability in order to affect tissue formation to a meaningful level in this model.

## Background

The spondyloarthropathies (SpA) form a class of joint diseases which specifically affect the vertebral column. Ankylosing spondylitis (AS), the prototypic form of SpA, is a chronic inflammatory arthritis of the primarily axial skeleton. One of the most debilitating aspects of AS is the progression from inflammation to bone formation, where inflammation is triggered by an unknown mechanism at the site of tendon and ligament attachments to bone, resulting in enthesitis, followed by the formation of bony projections (syndesmophytes) which ultimately may join and result in ankylosis. Inflammation can be well controlled in many patients by anti-TNF therapy, however ankylosis may still progress [1–4]. Although a recent Cochrane review determined that the effects of long term high-dose NSAID treatment on syndesmophyte formation was unclear [5], other recent studies have indicated chronic high-dose NSAID treatment [6–8] or long-term TNF blockade [3, 9] may be effective at slowing progression. No treatment to date has managed to halt or reverse radiographic progression in AS.

A major factor in the poor treatment options available for advanced AS is the lack of understanding of the molecular mechanisms driving disease progression. Several recent studies in both patients and mouse models have identified the Wingless (Wnt) pathway, key for bone development and homeostasis, as disturbed in AS. Sclerostin (SOST) is specifically expressed in osteocytes and chondrocytes providing skeletal tissue specific inhibition of Wnt signalling. Inactivating mutations in SOST which lead to increased Wnt signalling result in increased bone mass and bone strength, as demonstrated in both mouse models and human disease [10].

SOST’s tissue-specific expression makes it an attractive therapeutic target in bone disease. In patients with SOST-inactivating mutations, the only phenotypes which develop are a direct consequence of high bone mass [11]. Antibody therapies to SOST have recently successfully completed phase 2 clinical trials, demonstrating an increase in bone density and a decrease in bone turnover markers in osteoporosis patients [12, 13]. In AS, which exhibits excess bone formation and where dysregulated Wnt signalling has been established, enhancing SOST activity presents an intriguing therapeutic possibility. To date no studies have investigated treatment with exogenous SOST to inhibit bone formation. In this report we have tested the effects of treatment with recombinant SOST to reduce osteoproliferation in the proteoglycan-induced spondylitis (PGISp) mouse model of ankylosing spondylitis.

## Methods

### Ethics statement

All mice were kept in specific pathogen-free housing and fed a standard diet, with water ad libitum. The study was carried out in accordance with the Australian Code of Practice for the Care and Use of Animals for Scientific Purposes (7^th^ Edition). The protocol was approved by the University of Queensland Animal Ethics Committee (Permit Number: UQDI/PAH/293/12/NHMRC).

### Animals

Spondylitis was induced by injecting three month old female BALB/c IL-4^−/−^ mice with 2 mg human proteoglycan extract in 2 mg dimethyldioctadecylammonium (DDA) 4 times at two week intervals [14, 15]. Peripheral arthritis presented after the second injection and mice exhibited axial skeleton ankylosis as described previously [15, 16].

Recombinant SOST (rSOST) was kindly provided by Michaela Kneissel (Novartis Pharma AG, Basel, Switzerland). Human glycosylated sclerostin was produced in HEK cells and purified using a proprietary tag. Activity was confirmed by the manufacturer prior to supply. rSOST injections were initiated at week 6 after commencement of the study once all four injections of proteoglycan had been administered, and prior to the development of arthritis. Eight mice were injected subcutaneously daily for eight weeks with 2.5 μg of rSOST dissolved in Dulbecco’s PBS. The control group received only the initial proteoglycan injections. Peripheral disease was scored daily in all mice. Scoring was on a scale of 0–4 where 0–3 came from previously published methods [14]. A score of 4 was assigned when severe joint stiffness was apparent by greatly reduced flexion but little swelling in the rear limb joints. Due to difficulties in accurately ascertaining joint stiffness in front limbs, only rear leg scores are included in the analyses. Mice exhibiting no signs of peripheral disease were removed from the analysis (two mice). Bone density was measured using a PIXImus (GE Medical Systems Lunar, Madison, WI) as described previously [17]. Serum collection and bone density scanning were carried out immediately prior to the first rSOST injection and after four and eight weeks of rSOST injections. Mice were sacrificed after 8 weeks of rSOST injections for histological examination. Axial disease could not be scored longitudinally as the spine and pelvis cannot be imaged non-invasively with sufficient resolution to follow disease from early stages.

## Histology

### Histological scoring

Intervertebral joints in hematoxylin and eosin (H & E) stained sections were scored from 0–4, using a validated scoring system [18]. Only well sectioned joints were scored; these were defined as joints with a clear nucleus pulposus and annulus fibrosis, or in the case of diseased joints, the presence of square vertebrae. The average joint score was calculated for each section, in order to compare sections with differing numbers of joints.

### Immunohistochemistry

DAB immunohistochemistry of formaldehyde-fixed paraffin-embedded samples was performed as described previously [19]. Briefly, samples were rehydrated, blocked with appropriate sera, and then incubated with primary antibody. Goat anti-mouse SOST (AF1589, R & D Systems, Minneapolis, MN USA) at 1:100 was incubated for 1 h. Sections were then incubated with the appropriate biotinylated secondary antibody, followed by streptavidin conjugated tertiary antibody. Signal was developed using Cardassian DAB chromogen kit (Biocare Medical, Concord, CA USA) and sections counterstained with acid hematoxylin (Sigma, St. Louis, MO USA) for three minutes. Sections were rinsed then left to dry overnight before mounting in DPX mounting media (Sigma, St. Louis, MO USA).

## IHC quantification

### SOST

In PGISp mice, SOST + ve osteocytes were quantified as described previously [20]. Data was analysed as a percentage of SOST + ve osteocytes from the total number of osteocytes counted. Data analysis was performed using GraphPad Prism software, and a t-test used to identify differences between groups.

### Elisa

To assess the kinetics of rSOST stability in vivo, five IL4^−/−^ mice were injected with 2.5 μg rSOST and serum collected 15 min, 1 h, 4 h, 8 h and 24 h after the initial injection. Serum levels of SOST were measured by an ELISA which detected both human and mouse SOST (UCSN Life Sciences, Inc., Wuhan, China).

## Results

### rSOST injection

**Fig. 1:**
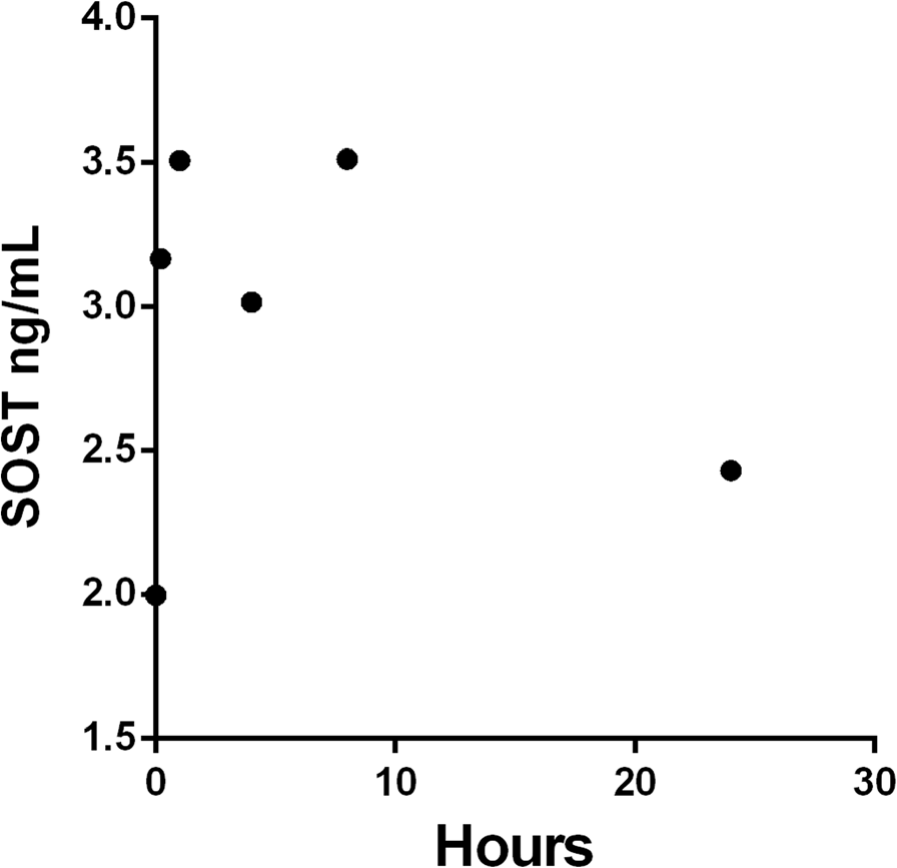
Elevation of SOST in serum. As observed using ELISA, SOST elevation in serum was detectable at all time points tested between 15 min and 24 h after rSOST injection

Rapid breakdown of subcutaneously administered rSOST might prevent the accumulation of an efficacious dose in the mouse. The bioavailability of rSOST in serum was assessed by ELISA over a 24 h time course (Fig. 1). The level of SOST was sharply elevated from 15mins after injection and remained elevated after 8 h (Fig. 1). This detected elevation probably reflects an increase in total circulating SOST caused by the injection of rSOST, rather than a secondary increase in circulating endogenous mouse Sost, since it is detected as early as 15 min after injection. Compared to the zero time point, SOST levels are still slightly elevated 24 h after injection, suggesting rSOST could accumulate with a daily injection regime.

### Circulating SOST levels

**Fig. 2:**
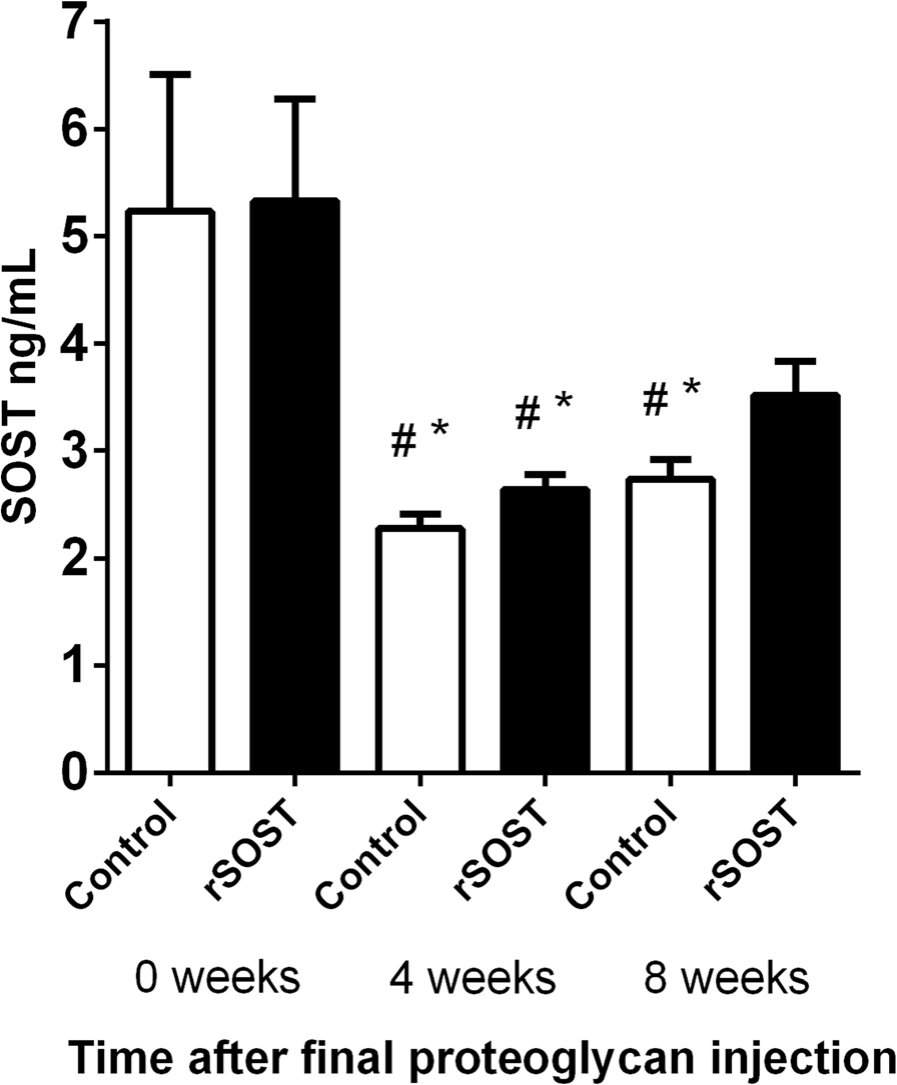
Serum SOST levels in control and rSOST treated mice. A decrease in SOST is apparent at both 4 and 8 weeks after the final proteoglycan injection (0 weeks). # = vs 0 week control, *p* < 0.05. * = vs 0 week rSOST, *p* < 0.05

After establishing that rSOST was detectably increased in serum for at least 8 h of the 24 h injection period we examined if repeated daily injections resulted in a systemic accumulation of SOST. Serum SOST levels were measured at the start of the study and after four and eight weeks of rSOST treatment. A sharp drop in serum SOST levels was seen 4 weeks after the final PG injection. There was a slight recovery in the control mice by 8 weeks and this was greater in the rSOST-treated mice but not significantly so. Previously we have shown that SOST expression in bones is reduced in PGISp mice [15]. Similarly, circulating levels of SOST are also reduced in PGISp mice both 4 and 8 weeks after their final proteoglycan injection, irrespective of rSOST treatment (Fig. 2).

### rSOST effects on disease progression and severity

**Fig. 3:**
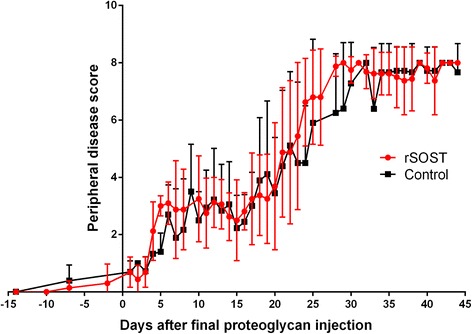
Impact of rSOST treatment on peripheral disease progression. There was no difference in the progression of peripheral disease between SOST treated and control mice. Scoring commenced prior to the first rSOST injection (Day 0). rSOST treated mice in red, control mice in black

In PGISp mice, peripheral arthritis precedes axial disease and can be scored by monitoring paw swelling and stiffness of rear leg joints [14]. Daily treatment with 2.5 μg of rSOST made no difference to the progression of peripheral disease (Fig. 3).

**Fig. 4:**
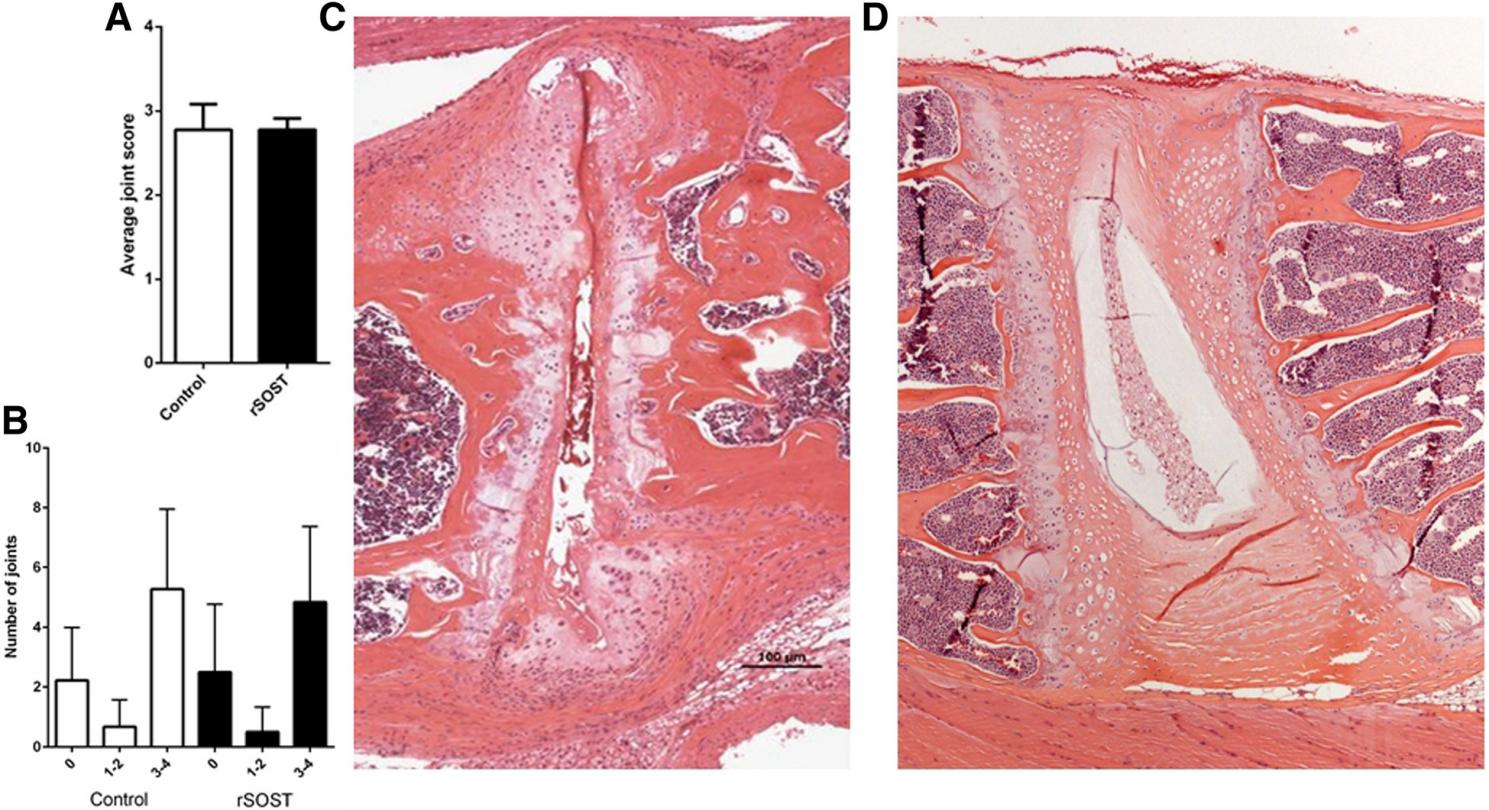
Axial skeleton histology. **a** No difference in histological scoring was observed between mice receiving rSOST (2.5 μg/day) and mice receiving no rSOST (Control) (*p* = 0.99). Joint scores were averaged for each section scored. **b** Intervertebral joints exhibiting inflammation (score 1 or 2) or ectopic tissue formation (score 3 or 4) were similar in number between control and rSOST treated mice. **c** A severely affected joint from a control mouse. The IVD has been completely destroyed with subsequent mesenchymal cell proliferation and cartilaginous matrix deposition with chondrocyte expansion evident. **d** Normal intervertebral joint, no signs of inflammation, disc destruction or excessive tissue production

Axial disease progression was assessed histologically. Scoring of intervertebral joints stained with haematoxylin and eosin showed that rSOST treatment had no effect on disease severity in the PGISp model (Fig. 4a, *p* = 0.99). The number of intervertebral joints exhibiting inflammation (score 1 or 2) or ectopic tissue formation (score 3 or 4) is similar between control mice and rSOST treated mice (Fig. 4b). Both rSOST treated and PGISp control mice had large numbers of severely affected intervertebral joints, with significant disc destruction and large-scale production of excess tissue (Fig. 4c). Not all joints in every mouse were affected; some appeared completely normal (Fig. 4d). This is a common feature of AS, and also of the PGISp mouse model [15]. rSOST treatment had no effect on the proportion of joints affected (data not shown).

### rSOST effects on bone mineral density

**Fig. 5:**
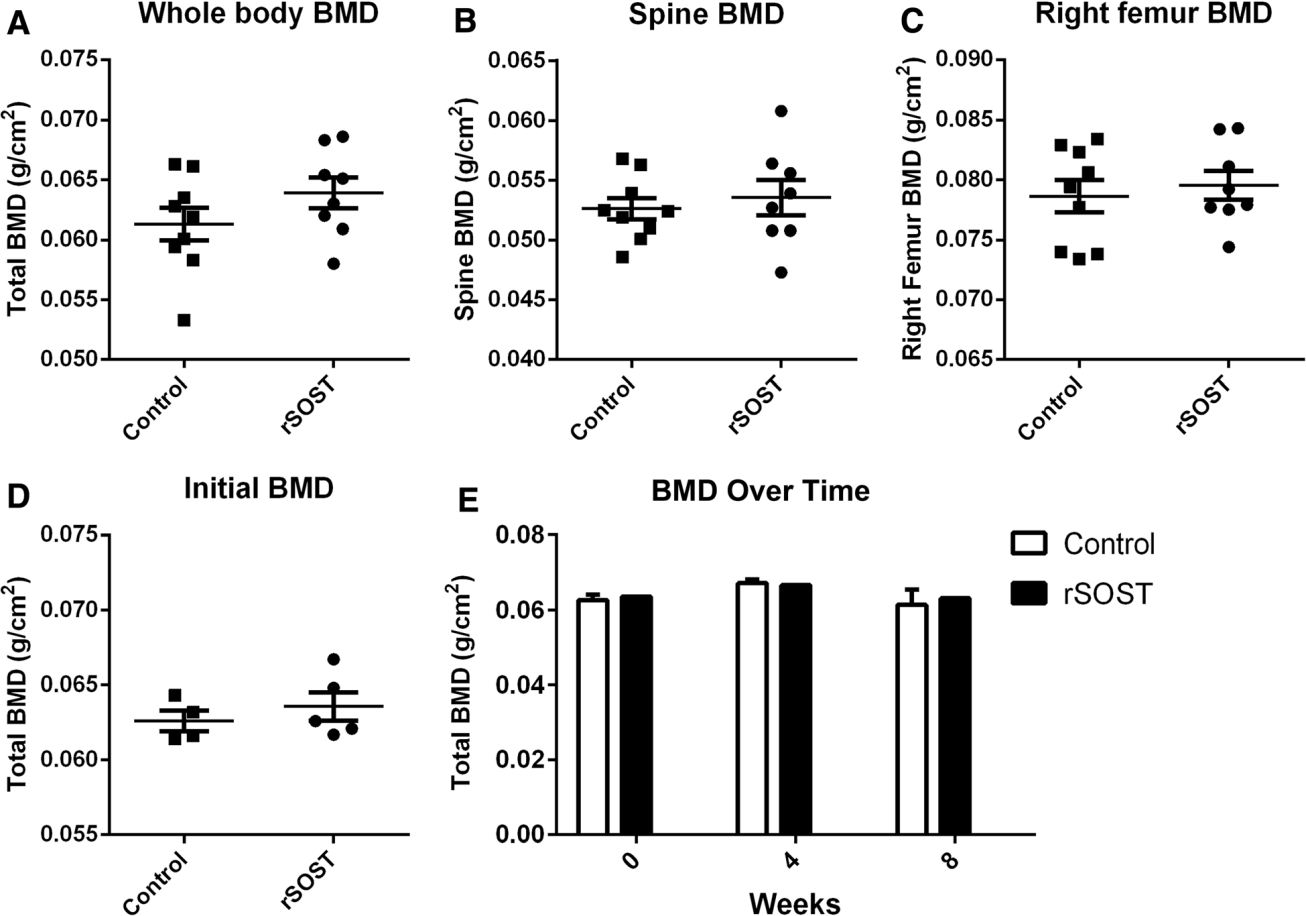
rSOST impact on bone density. No systemic or localized changes in bone density were observed after rSOST treatment. There was no difference in initial or final bone density between the three groups. **a** Whole body BMD at the termination of the experiment (*p* = 0.19). **b** Terminal BMD of spine and pelvis only (*p* = 0.59). **c** Terminal right femur BMD (*p* = 0.62). **d** Initial whole body BMD measurement after four proteoglycan injections (*p* = 0.46). **e** Whole animal BMD by treatment group at each time point (*p* = 0.7)

Altered levels of circulating SOST can result in systemic bone density changes, as seen in the SOST-transgenic mouse, where increased SOST levels lead to bone loss [11]. Bone density was measured to assess potential systemic bone loss. There was no difference in total body, spine or femur bone mineral density (BMD) between rSOST treated and control mice (Fig. 5a-d). There was also no difference with rSOST treatment at any of the time points examined (Fig. 5e). The ineffectiveness of rSOST injections on axial disease progression or BMD suggests the exogenous SOST is not affecting the skeleton.

### Wnt inhibitor expression

**Fig. 6:**
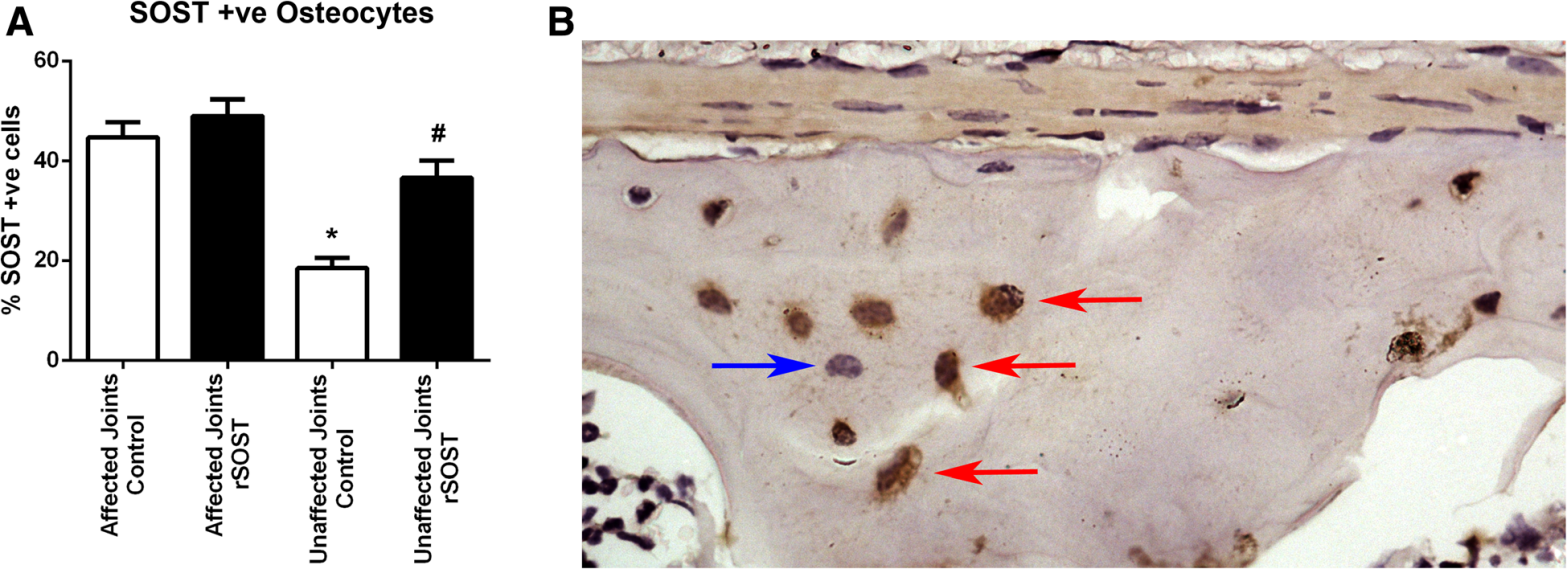
SOST expression in osteocytes. No difference in the percentage of SOST positive osteocytes in affected vertebrae between untreated and rSOST treated mice. **a** There was a decrease in SOST expression at unaffected joints, and a significant decrease in unaffected control mice compared to all other groups (* *p* <0.05). There was a significant increase in SOST positive cells in the unaffected joints of mice treated with rSOST in comparison to the unaffected joints of untreated control mice (# *p* < 0.05). **b** Example of SOST positive osteocytes (*brown staining, red arrows*). A negative osteocyte is also clearly visible (*purple staining, blue arrow*)

Immunohistochemistry was used to quantify SOST expression. SOST expression was observed in osteocytes and also in chondrocytes resident in calcified cartilage. There was no difference in the percentage of SOST positive osteocytes between rSOST treated and untreated control PGISp mice in severely affected joints (score = 4, Fig. 6a). However, in control PGISp mice, there was a significant decrease in the percentage of positive osteocytes in the unaffected joints compared to the affected joints (*p* = <0.05, Fig. 6a). There was also an increase in the percentage of positive osteocytes in the unaffected joints of rSOST treated mice in comparison to the unaffected joints of untreated control PGISp mice (*p* = <0.05 Fig. 6a). This suggests that rSOST treatment increases the number of SOST-positive osteocytes in unaffected joints but not affected joints, despite having no impact on the number of joints affected by disease.

## Discussion

No treatment currently available is able to reverse or significantly slow the excessive bone formation which occurs in AS. Recent studies have demonstrated that Wnt signalling appears to be up-regulated in AS evidenced by decreased levels of the bone-associated Wnt inhibitor SOST in both human and mouse studies [15, 21, 22]. Inhibiting Wnt signalling through SOST supplementation therefore presents a potential therapeutic target for the specific treatment of this bone formation. However, daily treatment with rSOST did not alter disease progression or severity in the peripheral joints or axial skeleton joints of PGISp mice.

There are a number of factors which may have contributed to the lack of efficacy of rSOST at inhibiting excess tissue formation in this study, including efficacy of a human recombinant SOST in mice, low stability of rSOST, failure of rSOST to reach joints, or insufficient dosage. Human SOST has previously demonstrated activity in a mouse system; the human SOST transgenic mouse, in which a bacterial artificial chromosome (BAC) containing the human *SOST* gene was inserted, is osteopenic [11]. In this model overexpression of human *SOST* was effective at blocking Wnt signalling, resulting in a marked decrease in osteoblast activity and thus bone formation.

Recombinant SOST degradation appeared to be relatively slow in PGISp mice, as circulating SOST levels were significantly increased 8 h after injection and remained elevated after 24 h. Further over an 8 week injection protocol it appeared that circulating levels of SOST were increasing suggesting an accumulation of the protein. This suggests that injection of rSOST might be a viable approach if therapeutic efficacy could be demonstrated. Alternatively, the circulating SOST increases might be due to increased endogenous expression. Unfortunately, the ELISA used did not differentiate between human and mouse SOST isoforms.

rSOST may have been bound to other proteins in the bloodstream which prevented it from exerting its actions at the joint. However, changes in SOST levels within the joints were observed. rSOST treatment increased the number of SOST positive osteocytes in unaffected joints. In vitro, application of recombinant human SOST results in decreased expression of SOST in human pre-osteocyte cells [23]. However, the same levels of recombinant human SOST caused increased expression of SOST in less mature cultures thus application of exogenous human SOST in our study may have influenced endogenous mouse SOST levels.

The failure of injected SOST to have any effect on bone formation in this experiment does not exclude SOST as a potential therapeutic in AS. There is strong suggestive evidence from both mouse models and human patients that regulation of SOST is an important factor in bone formation in AS. Markedly reduced SOST expression has been shown both in serum and biopsies from AS patients [21, 22]. High SOST levels occur in hTNFtg mice, as TNF-α stimulates SOST expression [24, 25] but if the mice are treated with Dikkopf-1 neutralising antibodies, which also results in reduced levels of SOST, spondylitis occurs [24]. Additionally, SOST expression is downregulated in the intervertebral joints in proteoglycan induced spondylitis (PGISp) mice [15]. SOST may need to be further stabilized or targeted to the bone to increase its potency as a treatment. SOST’s bone specificity in non-pathological situations comes from its specific expression in osteoblasts and osteocytes rather than it having any endogenous bone-targeting properties. One possible strategy is to complex SOST with a bone-targeting moiety, such as a bisphosphonate, to allow the specific accumulation of SOST in the bone. This approach of attaching a therapeutic to a bisphosphonate for bone targeting has previously proven successful in a mouse model of metastatic breast cancer [26].

Alternatively, the attachment of an FcD_10_ motif to a recombinant human alkaline phosphatase protein has shown success in the treatment of hypophosphatasia [27, 28]. The FcD_10_ motif contains the human IgG_1_ Fc domain and a deca-aspartate motif. This motif created a strong affinity for hydroxyapatite while not reducing the activity of the enzyme. This targeting strategy has been successful in ameliorating hypomineralization in hypophosphatasia patients where direct infusion or injection of the alkaline phosphatase enzyme have previously failed [29].

This is the first study to report the injection of rSOST in a mouse model. There have been very few reports of in vivo studies targeting the skeleton utilising recombinant proteins. The dosage of 2.5 µg is similar to that used in previous studies. The lack of efficacy of rSOST to alter the disease course in treated mice may have therefore been a contributed to by an insufficient dosage administered. Future studies should investigate the impact of higher dosages, including safety and toxicity profiling.

Encouragingly, a recent study examining Wnt inhibition through inhibiting β-catenin function in murine systemic sclerosis, another mouse model of rheumatic disease, demonstrated efficacy in reducing and reversing the primary disease symptom of fibrosis. Despite the administration of the Wnt inhibitors systemically, no other side effects such as hair loss or gut abnormalities were seen [30].

The aim of this study was to specifically focus on the potential role of Wnt signalling inhibition in a mouse model of AS. Current knowledge suggests that Wnt signalling will play a role in AS through the osteoproliferative phase of the disease. The PGISp mouse is a good choice for such a study as it has been well established that this model exhibits axial osteoproliferation similar to the human condition. However there are a number of differences between the PGISp mouse model and AS. Disease in PGISp mice favours females rather than males and there is no strong evidence for a significant role for HLA-B27. A different model could be used to examine the effect of rSOST on bone formation, such as transgenic HLA-B27 rats which spontaneously develop spondylitis and also exhibit axial osteoproliferation [31]. Alternatively, a non-AS model could be used, such as the spinal cord injury induced model of neurological heterotopic ossification where local inflammation leads to ectopic bone formation [32]. Application of exogenous rSOST in these models may lead to additional insights into the role and importance of Wnt signalling in pathological bone formation and how this may be manipulated in a therapeutic setting.

## Conclusions

Further studies are required to fully determine if SOST is an appropriate therapeutic to inhibit bone formation in AS. Although not disease-modifying, rSOST treatment did appear to regulate SOST levels in the joints suggesting biological activity. The PGISp mouse model is a good model of inflammatory bone formation in AS particularly to examine therapies targeting Wnt signalling. Downregulation of SOST in this model phenocopies the downregulation of SOST previously reported in human patients. Further dose response studies are required and SOST may require modifications to improve its bone targeting ability in order to affect tissue formation to a meaningful level in this model.
